# Drug Repurposing in Medical Mycology: Identification of Compounds as Potential Antifungals to Overcome the Emergence of Multidrug-Resistant Fungi

**DOI:** 10.3390/ph14050488

**Published:** 2021-05-20

**Authors:** Lucie Peyclit, Hanane Yousfi, Jean-Marc Rolain, Fadi Bittar

**Affiliations:** 1 Aix Marseille Univ, IRD, APHM, MEPHI, 13005 Marseille, France; lucie.peyclit@ap-hm.fr (L.P.); hanane.yousfi@univ-evry.fr (H.Y.); jean-marc.rolain@univ-amu.fr (J.-M.R.); 2 IHU Méditerranée Infection, 13005 Marseille, France

**Keywords:** drug repurposing, antifungals, repositioning, yeasts, emerging fungi, multidrug resistance, therapeutic alternatives, new targets, *Candida auris*, *Aspergillus* spp.

## Abstract

Immunodepression, whether due to HIV infection or organ transplantation, has increased human vulnerability to fungal infections. These conditions have created an optimal environment for the emergence of opportunistic infections, which is concomitant to the increase in antifungal resistance. The use of conventional antifungal drugs as azoles and polyenes can lead to clinical failure, particularly in immunocompromised individuals. Difficulties related to treating fungal infections combined with the time required to develop new drugs, require urgent consideration of other therapeutic alternatives. Drug repurposing is one of the most promising and rapid solutions that the scientific and medical community can turn to, with low costs and safety advantages. To treat life-threatening resistant fungal infections, drug repurposing has led to the consideration of well-known and potential molecules as a last-line therapy. The aim of this review is to provide a summary of current antifungal compounds and their main resistance mechanisms, following by an overview of the antifungal activity of non-traditional antimicrobial drugs. We provide their eventual mechanisms of action and the synergistic combinations that improve the activity of current antifungal treatments. Finally, we discuss drug repurposing for the main emerging multidrug resistant (MDR) fungus, including the *Candida auris*, *Aspergillus* or *Cryptococcus* species.

## 1. Introduction

Most public health organizations, including the World Health Organization, do not have a fungal infection surveillance program, despite the fact that invasive fungal infections present a high mortality rate worldwide, often exceeding 50% [1,2]. Fungal infections have long been poorly documented and recognized, perhaps due to the need to treat other severe and serious bacterial and viral infections. However, mycoses should no longer be ignored.

The signs and symptoms of fungal infection appear during antibiotic therapy, particularly due to opportunistic fungal agents. In particular, invasive fungal infections affect patients with compromised immune systems, such as those with hematologic malignancies, HIV infection, chemotherapy treatments, etc. [3,4]. In addition, other factors such as the ageing of the population, which is susceptible to these opportunistic infections and improvements in diagnostic methods have led to their increasing prevalence in hospitals. A significant number of fungal agents, including yeast and yeast-like species such as *Candida* spp., *Cryptococcus* spp. and molds such as the *Aspergillus* species, complicate clinical management, with a variety of symptoms, prevalence and clinical outcomes [5]. Moreover, fungal infections can also occur in healthy people, so it is difficult to control their spread [6]. Therefore, greater consideration should be given to monitoring fungal infections [7,8,9].

To deal with these fungal infections, there are only four main therapeutic classes currently used in clinical practice, namely polyenes, azoles, echinocandins and flucytosine. Although these drugs remain active, they display several limitations that complicate their routine use including off-target toxicity, drugs interaction, clinical failure and long-term treatment [10]. Furthermore, the emerging resistance to antifungals and the poor clinical response of many isolates to antifungal therapy make this an even greater public-health concern. For example, previous exposure to an antifungal agent such as fluconazole has been shown to increase the risk of fluconazole-resistant *Candida* infections in immunocompromised patients [11]. Antifungal resistance remains a critical global problem, although it may vary depending on the species, geography and available therapeutic alternatives [5]. Some species are known to be more resistant than others, leading to treatment failure; these include *Candida* pathogens (*Candida glabrata*, *Candida krusei*, *Candida lusitaniae* and the very newly emerging yeast: *Candida auris*), some cryptococcal species and opportunistic *Aspergillus* or *Fusarium* species associated with immunocompromised hosts [5]. The aforementioned species commonly exhibit high intrinsic antifungal resistance profiles, sometimes to different classes of antifungals as is the case with almost all *Fusarium* spp. to triazoles, 5-fluorocytosine and echinocandins [5].

Despite great efforts made internationally to deal with antibiotic resistance by reducing the inappropriate consumption of antimicrobials, this does not yet extend to the use of antifungal agents. In reality, antifungal resistance may arise from fungicide use in agriculture, as described in a recent study in 2017, where plant bulbs were found to be positive for triazole-resistant *Aspergillus fumigatus*, rather than from clinical use [12]. Furthermore, antifungal therapies are mostly given to immunocompromised patients or those in intensive care units (ICUs), when treatment is unavoidable, rather than in preventive use in community medicine. Demers et al. described the impact of the heterogeneity of a single gene (MRR1), found in different *C. lusitaniae* subpopulations in an azole-naïve cystic fibrosis patient, on the level of fluconazole resistance, highlighting other indirect factors involved, such as the host immune system and coinfecting bacteria [13]. Therefore, reducing the consumption of antifungals may not be the only solution to improving the difficult management of invasive fungal diseases.

The development of new antifungal drugs represents a major challenge for the pharmaceutical industry, since fungi are eukaryotic organisms and have a close evolutionary relationship with their human hosts [14]. In addition, the pharmaceutical industry is no longer interested in developing and marketing new antimicrobials, including antifungals [1,15]. Indeed, the drug development process remains very expensive, time-consuming and risky due to many factors [16]. Given the difficulties of treating invasive fungal infections, consideration should be made to use efficient alternative strategies to implement immediate and appropriate measures. New targets involving enzymes and other metabolic pathways, or new formulations/generations are under development to broaden the spectrum of antifungal activities and to potentially overcome current resistances [17,18,19,20]. New antifungals can also be found in natural products or plant extracts, inspired by traditional medicine and aromatherapy [21,22]. Indeed, various essential oils showed in vitro efficiency against clinical yeast or fungi [23,24,25]. Preclinical studies to determine human toxicities, pharmacodynamics and clinical trials must continue [21,25]. At the same time, the strategy of drug repurposing (also called drug repositioning) consists of identifying drugs known to be effective for another indication than that for which they are marketed [15]. This strategy has gained in popularity in recent years and has already proven to be effective, particularly in oncology, cardiology and Alzheimer’s disease. These reinvestigated drugs have already completed preclinical trials, main human toxicities are well known, and the research and development process can be, considerably, reduced allowing for lower investment costs and faster potential clinical use [15,26]. However, the setting up of this strategy is not a straightforward matter as it does not exclude carrying out further clinical trials before the simple repositioning of a given drug, but it must also initially deal with the intellectual property rights, regulatory/authority process, license grant, pricing, patient’s acceptance, marketing strategy and commercialization in order to avoid any failure at the development stage [27].

The aim of this review is to report on the non-antifungal drugs that may be active against the most common emerging multidrug-resistant (MDR) fungi in human pathology. We first summarize the antifungal compounds currently used for clinical therapies and their main mechanisms of resistance and then report on alternative drugs used to treat fungal infections that have been reported in the literature so far. Finally, we address different drugs that have a potential for repurposing to treat the main difficult to treat fungi.

## 2. Current Antifungal Agents and Their Mechanisms of Resistance

**Figure 1:**
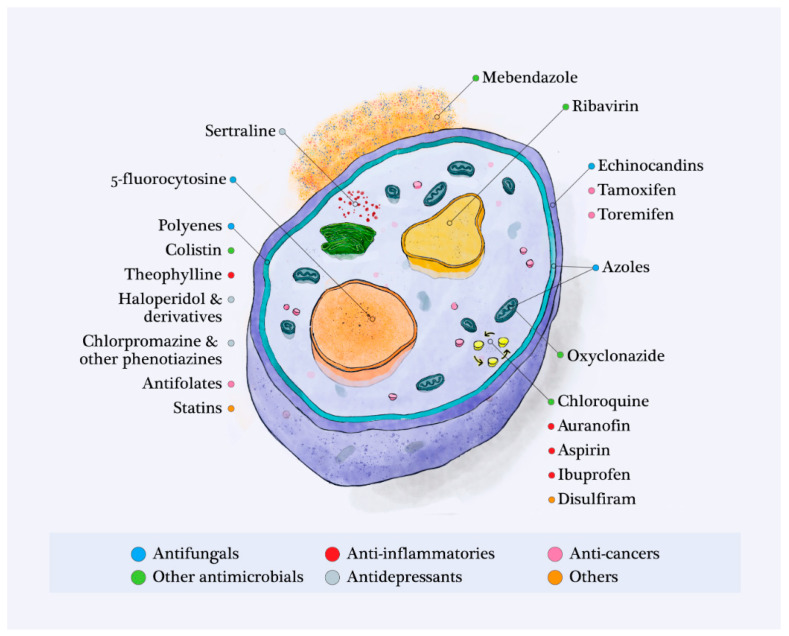
Therapeutic targets in the fungal cell of the compounds listed in the review: known antifungals and molecules that have a potential for repurposing as antifungals.

Since their first and progressive discovery in the mid-20th century, systemic antifungals have improved the management of many invasive fungal infections. The spectrum of antifungals and their mechanisms of action are diverse, since they act on different structures in the fungal cell; we describe them below (Figure 1).

The first antifungal agents, polyenes, were developed in 1950s for clinical use and have a large spectrum of activity against yeast and filamentous fungi. Polyenes are macrocyclic organic molecules from a soil actinomycete, *Streptomyces nodosus* [28]. The two most clinically relevant members of this class are topical nystatin and intravenous amphotericin B. Both bind to a sterol moiety, ergosterol, on the fungal cell membrane, disrupting cell permeability and leading to cellular lysis. Nystatin is not effective against dermatophytes but is effective against the *Candida* species [29]. Liposomal amphotericin B has been developed to allow the administration of higher doses with less nephrotoxicity (fewer side effects) to mammalian cells.

Resistance to polyenes is mainly related to changes in the lipid structure of the membrane and subsequently a modification in its fluidity and absorbency. The principal altered effects concern enzymes that are involved in the synthesis of ergosterol. Deficiencies in the *ERG2* and *ERG3* genes, which code for the isomerase of C-8 sterol and delta-5,6-desaturase, induce modifications in membrane sterols. The quantity of ergosterol is modified, consequently affecting polyene activity. Boosted activity of catalase, an antioxidant enzyme that decreases oxidative injury, represents another polyene-resistance mechanism. However, intrinsic amphotericin B resistance frequently described in some *Aspergillus* spp. strains not only include the alteration of the ergosterol pathway but the signaling pathways, such as those described in *A. terreus* [30] or increased enzymatic activity of the peroxidase and superoxide dismutase in *A. flavus* [31] (Table 1).

Discovered in 1980s, azoles now represent the best conventional antifungal agents for medical treatment. Azole compounds have had a major impact on the treatment of invasive fungal infections over the last 35 years. The use of the first available azoles, imidazoles including ketoconazole, miconazole and clotrimazole, was primarily restricted to the treatment of superficial fungal infections. These compounds were substituted by the first-generation of triazoles, such as fluconazole and itraconazole, to broaden the range of application. Later, the search for new antifungals was intensified to overcome some efficiency limitations and to prevent emerging resistant pathogens. The second generation of triazoles (voriconazole, posaconazole, efinaconazole and isavuconazole) were developed with an extended spectrum of activity [32,33]. The final target of these drugs is ergosterol from the cell membrane. They inhibit lanosterol-14α-demethylase in mitochondria, which interferes with the synthesis of the membrane ergosterol. Each azole has a different affinity in its inhibition of lanosterol, which may explains the differences in spectrum of activity among azole agents [34], but all have a strong inhibitor effect on the CYP450 enzyme system, which is responsible for many drug interactions [35]. The global HIV epidemic led to the widespread and significant use of fluconazole to treat oro-oesophageal candidiasis in HIV-infected patients and fluconazole-resistant *Candida* strains were later widely reported in these patients [34,36].

Four mechanisms of resistance have been demonstrated so far: (i) activation of the efflux pumps due to an overexpression of membrane-associated transporters encoded by the gene families of transporters (CDR and MDR) reduces azole plasmatic concentrations [30,34]; (ii) azole agents cannot, qualitatively, bind to their enzymatic target to target changes induced by a mutation in *ERG11* gene, which encodes for the lanosterol-14α-demethylase [33,34]; (iii) some strains induce the overexpression of ERG11 as a compensatory mechanism and increase the intracellular concentration of this protein. Thus, an increasing concentration of a given azole agent is needed to remain efficient. This resistance mechanism involves quantitative changes by upregulating the target enzyme [30,34]; (iv) some strains, with a mutation of the ERG3 gene, developed a pathway bypassing fungal membrane biosynthesis by replacing ergosterol with 14α-methyl-fecosterol and preventing the accumulation of a toxic product: 14ɑ-methyl-3,6-diol. This alternative route of ergosterol biosynthesis maintains both the function of the fungal membrane and the resistance to azoles [33] (Table 1).

Mannan, chitin and α- and β-glucans are the main compounds of the fungal cell wall. Drugs belonging to the echinocandins target one of these components and act as antifungals, inhibiting β-(1,3)-glucan synthetase leading to a depletion of β-(1,3)-glucan, an essential component for the structuring and function of the cell wall [33]. Echinocandins (caspofungin, anidulafungin and micafungin) are semisynthetic cyclic lipopeptides derived from natural products. They present a reserve supplement to the arsenal of drugs available to treat invasive fungal diseases with a fungicidal action against the *Candida* species and a fungistatic activity against the *Aspergillus* species [37]. 

*Candida* resistance to echinocandins has been related to several mutations in a hot-spot region of the *FKS1* gene, which encodes for a subunit of echinocandin target, resulting in a lower affinity between the antifungal and its target [5,30] (Table 1).

A pyrimidine analogue, 5-fluorocytosine (5-FC), was developed in 1957 as an antimetabolite. Without any potential use as an anticancer treatment, it was used to treat fungal infections. Once 5-fluorocytosine enters the fungal cell, enzymes convert it in compounds to be incorporated in the synthesized RNA. This disrupts the protein synthesis of the affected fungi. It is also converted as a potent inhibitor of thymidylate synthase, which interferes in DNA synthesis and nuclear division [33]. It is always combined with other drugs, in association with azoles or amphotericin B, due to the high prevalence of intrinsic resistance in many fungal species and to the rapid development of resistance in yeast [33,34]. The primary resistance was about 10% in the *Candida albicans* strains [30]. Resistance to 5-FC may be due to mutations in *FUR1* or in the genes *FCY1* and *FCY2* leading to defects in flucytosine metabolism [30] (Table 1).

Finally, it is notable that, besides the limited number of available antimycotic agents and the burden of antifungal resistance (described above), toxicities and drug–drug interactions of antifungals, the treatment failure due to clinical resistance is frequently reported in invasive mycoses [34]. In fact, in vitro antifungal susceptibility testing does not guarantee the success of in vivo treatment. Many factors affecting the infected patient, the responsible fungal strain and the prescribed antifungal agent, which have been well reviewed elsewhere [34], may explain this clinical resistance.

## 3. Non-Antifungal Drugs Identified as Having a Potential Antifungal Activity against Invasive Fungal Strains

Given the rapid evolution of resistance to antifungal drugs and the high prevalence of mycoses in clinical settings due to the increasing number of human immunodeficiency cases and/or as the result of improved fungal diagnosis, there is an urgent need to improve the efficacy of current treatments and to develop new therapeutic strategies. It is worth considering innovative approaches [5] or, simply, associations of existing drugs, which could be a promising approach to extending the use of current antifungal agents. Indeed, drug combination resulting in a synergistic activity has the potential to impede the evolution of drug resistance through employing several mechanisms or targets [15]. However, this should not be considered as the only “miracle” solution because interactions can be indifferent and combinations can be unsuccessful [38].

Drug repositioning or repurposing allows for new indications for previously approved drugs that are already marketed for other medical reasons. This approach offers many benefits over de novo drug development. Previously established pharmacokinetic and pharmacodynamic profiles and toxicity data allow for faster and cheaper development of repositioned molecules. Consequently, clinical use may be considered to overcome the rapid emergence of resistant fungi and outbreaks [15,39]. In this paper, we reported on molecules that have been found to be active in vitro or even in vivo against fungal agents, according to their initial therapeutic class (Figure 1 and Table 2).

### 3.1. Antimicrobials Apart from Antifungals

Polymyxins including colistin and polymyxin B are peptide antibiotics and target the outer membrane of Gram-negative bacteria [40]. Tested against MDR yeasts and molds, polymyxins showed an antifungal activity with minimum inhibitory concentrations (MICs) ranging from 16 to 128 µg/mL [41]. In particular, a fungicidal effect was described with colistin against *C. albicans*, *Cryptococcus neoformans* and *Rhodotorula mucilaginosa*. The mechanism of action was similar to the bacterial mechanism, by inducing membrane damage to MDR-*C. albicans*, as observed under fluorescent microscopy [41]. Using a checkerboard microdilution assay, synergistic activity was revealed with colistin-amphotericin B and colistin-itraconazole, against MDR *C. albicans* and *Lichtheimia corymbifera* strains [41]. The colistin-azoles combination has also been reported more recently in strains showing low susceptibilities to fluconazole. Bibi M. et al. confirm that colistin binds to the lipids of fungal membranes and works in relation to an ergosterol depletion level due to the previous action of azoles [42].

**Table 2:** Drugs with reported in vitro antifungal activities. NR: not reported.

	Drug	First Indication	Antifungal Activity	Activity Range	Antifungal Mechanism of Action	References
**Antimicrobials**	**Polymyxins** *Colistin* *Polymyxin B*	Gram-negative bacterial infections	*C. albicans*	16–128 µg/mL	Membrane damages on *Candida albicans*	[41]
			*C. neoformans*			
			*R. mucilaginosa*			
			*S. apiospermum*			
			*L. prolificans*			
			*F. oxysporum*			
			*F. solani*			
			*R. oryzae*			
	**Ribavirin**	Hepatitis C	*C. albicans*	0.37–3.02 µg/mL	Disruption of vacuoles function of *C. albicans* strains	[41,43]
			*C. tropicalis*			
			*C. parapsilosis*			
	**Oxyclozanide**	Animal parasitosis	*C. albicans*	16–32 µg/mL	Uncoupling the mitochondrial electron transport from phosphorylation and changing the mitochondrial membrane potential	[44]
	**Chloroquine**	Malaria	*C. neoformans*	3.19 µg/mL	Iron deprivation	[45]
				(10 µM)		
			*C. albicans*	31.2–250 µg/mL	Inhibition of ergosterol biosynthesis	[46]
			*S. cerevisiae*	NR	Growth inhibition via blocking thiamine transportation	[47]
	**Mebendazole**	Helminthiasis	*C. neoformans*	92.5 ng/mL	Morphological alterations by reducing capsular dimension	[48]
			*C. gatti*	(0.3125 µM)		
**Anti-inflammatory**	**Auranofin**	Rheumatoid arthritis	*C. albicans*	0.25–16 µg/mL	Action on reactive-oxygen-mediated cell death	[49,50]
			*A. fumigatus*			
			*S. apiospermum*			
			*L. prolificans*			
			*C. neoformans*			
	**Aspirin**	Inflammation	*Cryptococcus* spp.	1–10 mg/mL	Stress induction via ROS-mediated damage	[51,52]
	**Ibuprofen**		*Candida* spp.			
	**Theophylline**	Asthma, COPD	*Candida* spp.	1.4–1.8 mg/mL	Membrane damages by ionic and ergosterol modifications	[53]
**Antipsychotics**	**Haloperidol**	Psychosis	*C. albicans*	<4 µg/mL	Possible action on GPCRs, mediators of signals across the cell membrane	[54,55]
	**Trifluperidol**		*C. neoformans*			
	**Sertraline**	Depression	*C. neoformans*	2–6 µg/mL	Inhibition of protein synthesis	[56,57]
			*Lomentospora prolificans*			
			*Scedosporium* spp., *Fusarium* spp.	8–32 μg/mL		
			*Paecilomyces* spp*., Alternaria* spp. and *Curvularia* spp.			
	**Chlorpromazine**	Schizophrenia	*Candida* spp.	1–16 µg/mL	Possible modifications of membrane	[58,59]
			*C. neoformans*			
			Filamentous fungi: *Aspergillus* spp., *Scedosporium* spp., *Pseudallescheria* spp. and			
			Zygomycetes			
**Anticancers**	**Tamoxifen**	Breast cancer	*Candida* spp. *C. neoformans*	8–64 µg/mL	Prevention of proteins calmodulin from binding to calcineurin, cell lysis and alteration of fungal development	[60,61,62]
	**Toremifene**				Disturb the cell wall integrity via interaction with Ccr1	
**Others**	**Disulfiram**	Alcoholism	*Candida* spp.	1–16 µg/mL	Chelating metals Inhibition of multidrug transporter implicated in drug resistance	[63,64]
			*C. neoformans*			
			*Aspergillus* spp.			

Other non-antifungal agents with bacterial activity have demonstrated a synergy with antifungals, as described by Rossato et al. [65]. For example, erythromycin with amphotericin B showed no toxic in vivo effect and could be a promising novel combination in invasive antifungal therapy [66].

Ribavirin, a purine nucleoside analogue, displays a broad-spectrum activity against many RNA and DNA viruses. Ribavirin is used to treat hepatitis C virus in combination with interferon-α [67]. Tournu et al. identified ribavirin as a potential *C. albicans* disrupting agent of vacuole, which is essential to yeast pathogenicity [43]. Based on this, we demonstrated the fungistatic activity of ribavirin against MDR *C. albicans* and fungicidal activity against *C. parapsilosis*. MICs largely ranged from 1.56 to 12.5 μmol/L (0.37–3.02 µg/mL) against *C. albicans*, *C. parapsilosis* and *C. tropicalis*. Synergistic activity was also reported when the antiviral agent was combined with either amphotericin B, fluconazole or itraconazole, against MDR *C. albicans* and was thus proposed to be further investigated for clinical use [68].

In terms of antiparasitic (anthelmintic) drugs, the activity of oxyclozanide, a halogenated salicylanilide, was demonstrated against *C. albicans* isolates, including ones that were resistant to azole and echinocandin. This anthelmintic agent seems to alter/disturb the mitochondrial oxidative phosphorylation function and thus its ability to use the nonfermentable carbon sources by disrupting the mitochondrial membrane potential [44]. Oxyclozanide is widely used as an antiparasitic veterinary drug against the liver fluke *Fasciola hepatica* [69], and has been studied for antibacterial properties against colistin-resistant Gram-negative bacilli infections [70]. Pic et al. showed that oxyclozanide inhibited, at 58% and 99%, the growth of *C. albicans* at a concentration of 10 and 100 μM, respectively. These concentrations are comparable to the therapeutic dose used for an ovine weighing 45 kg [71], however its repurposing strategy in human therapies requires further and careful data on pharmacokinetics/pharmacodynamics and/or in vivo therapeutic assays, as this drug has not yet been used in humans.

The first and main antimalarial drug, chloroquine, is able to be used in different indications as it has many effects on inflammatory responses, metabolic process, the immune system and infections [72,73]. Weber et al. described that chloroquine treatment of macrophages infected with cryptococcal cells led to the formation of iron complexes inducing the death of *C. neoformans* [45]. Chloroquine has also been shown to inhibit thiamine transporters in the yeast *Saccharomyces cerevisiae*, linked to glucose metabolism [47]. It can also damage fungal morphogenesis due to an anormal synthesis of ergosterol in drug-resistant *C. albicans* strains [74].

Benzimidazoles such as mebendazole, albendazole, flubendazole and triclabendazole are broad-spectrum anthelmintic drugs. Joffe et al. demonstrated the efficacy of benzimidazoles in inhibiting the growth of *C. neoformans*, especially mebendazole and flubendazole [48]. Mebendazole has been suggested to be repurposed as an anticryptococcal drug because it can efficiently penetrate the blood–brain barrier in animal models [48,75].

### 3.2. Anti-Inflammatory Drugs 

Recently, Ogundeji et al. reported that aspirin and ibuprofen can control the growth of cryptococcal cells, with a high susceptibility of *C. neoformans* strains. Ibuprofen had a greater inhibitory effect than aspirin on all 10 *Cryptococcus* spp. tested strains at various drug concentrations [51]. The effects of ibuprofen seem to be dose-dependent; at high concentration (10 mg/mL), *Candida* cells are killed whereas at lower concentration (5 mg/mL), the drug was fungistatic [52]. In addition, synergistic outcomes were observed between ibuprofen and fluconazole or amphotericin B in *Cryptococcus* spp. and *Candida* spp., with fractional inhibitory concentration (FIC) < 0.5 [51,52,76].

Auranofin inhibits several inflammatory pathways and has been used since 1985 as an antirheumatic drug. It has already been found to be effective to treat bacterial infections [77] but Wiederhold et al. showed that auranofin also displayed an activity against various yeast and molds such as *A. fumigatus*, *Scedosporium apiospermum* and *Lomentospora prolificans* [49]. Although auranofin MICs were sometimes higher than those of the reference treatment (i.e., fluconazole), these concentrations could be easily achieved in patients’ blood treated with the usual therapeutic dose [49]. Therefore, auranofin, along with its activity on biofilms [78], could be used as a promising antifungal treatment [54,66]. However, due to its immunosuppressive action, this drug should be carefully investigated prior to administration as an antifungal agent, given that fungi-infected patients are commonly immunocompromised.

Finally, theophylline is generally used for asthma or chronic obstructive pulmonary disease (COPD) and has been proposed to treat candidiasis as it had effects on cell membrane integrity [53].

### 3.3. Antipsychotic Drugs

Oral antipsychotic drugs are often used in routine clinical practice, so their side effects and toxicity are now well known. Recently, haloperidol and trifluperidol were described for their antifungal activity against *C. albicans* (with MICs values < 4 µg/mL) or against *C. neoformans* [55,79]. The authors demonstrated that the two antipsychotics had a similar effect to fluconazole, acting on the yeast membrane [54]. The combination of a haloperidol-derivative with the antifungal posaconazole displayed a 16-fold reduction in MIC values for both the azole agent and the antipsychotic, compared with each drug alone (from >32 and >128 to 2 and 8 µg/mL respectively) with a fractional inhibitory concentration index (FICI) of 0.13 [79]. Strong synergies were also observed against *C. glabrata* and *Aspergillus terreus* [79].

Sertraline, the most frequently prescribed antidepressant, has been reported to be fungicidal against *C. neoformans*, with MICs ranging between 2 and 6 µg/mL [56,80,81] but also against emerging fungi [57,82]. Moreover, in vivo sertraline antifungal activity was tested, in murine models of cryptococcosis, showing a reduction in the fungal burden [56]. The combination of this compound and azoles or amphotericin B showed efficiency against various *Cryptococcus* spp. strains [83,84]. Of the 53 tested isolates, 31 were affected by the synergistic combination sertraline-fluconazole (FICI ≤ 0.5) [83]. However, sertraline has shown some antagonist effects with fluconazole against *Candida* strains [56].

Despite many in vitro, in vivo and human studies showing the efficiency of sertraline as antifungal, disparities appear in the clinicals studies [80,85]. In 2019, a clinical trial of 486 participants testing sertraline as an adjunctive treatment to IV amphotericin B and oral fluconazole to treat HIV-associated cryptococcal meningitis did not significatively improve survival, but did reveal a similar fungal clearance rate between groups [85]. In 2020, another randomized trial testing sertraline as pre-emptive therapy was stopped without a final conclusion due to the severe side effects of sertraline that were observed [86].

Chlorpromazine and trifluoperazine, dopamine antagonists, are used to treat schizophrenia. They are largely reported as having antibacterial and antifungal effects [58,59,87] and Vitale et al. confirmed the in vitro antifungal activity against difficult-to-treat filamentous fungi such as *Aspergillus* species (*A. fumigatus*, *A. ustus* and *A. terreus*), zygomycetes (*Absidia corymbifera*, *Rhizopus oryzae* and *R. microspores*) and *Scedosporium* species (*S. apiospermum* and *S. prolificans*) [58]. Phenothiazines including chlorpromazine appear to act on the fungal membrane but this needs further consideration [58]. The use of the combination must be advised to avoid resistance, to have greater efficacy and a less toxic effect: for example, chlorpromazine-amphotericin B against *C. neoformans* [84] or *Candida* species [59] demonstrated an interesting synergism profile.

### 3.4. Anticancer Drugs

Anticancer drugs may represent an important source of potential repurposing drugs. Indeed, as they often work on the basic metabolism pathways of eucaryotic and human cells such as the DNA replicating pathway, yeast cells may also be affected. Various anticancer drugs have been seen to be efficient in vitro against yeast growth [54,88]. Butts et al. demonstrated the fungicidal activity of tamoxifen and toremifene, two estrogenic receptor antagonists, against *C. neoformans* within macrophages, where the main pathogenesis of this organism happens [60]. Tamoxifen is described as an inhibitor of calmodulin. In this way, tamoxifen-treated yeasts showed cell lysis and an alteration of fungal development [61,89]. Recently, the interaction between tamoxifen and its target Ccr1 has also been described as causing the disruption of cell wall integrity [62].

However, administration of anticancer molecules to patients who are often immunocompromised should only be taken after careful consideration due to the numerous side effects that these compounds can cause, including immunosuppression [90]. A bypass solution via a synergistic association could circumvent this limitation. Interestingly, both compounds were synergistic in vitro with amphotericin B and fluconazole against cryptococcal cells [60]. In addition, in vivo candidiasis was cured, in a murine model, by the administration of 200 mg/kg of body weight per day of tamoxifen [61]. Since then, a randomized phase II clinical trial of tamoxifen as an adjuvant to the gold standard therapy for cryptococcal meningitis is in progress [91].

Antifolates as inhibitors of purine synthesis have also been reported to be effective agents against yeast development by reducing the quantity of ergosterol [65].

### 3.5. Other Approved Drugs

Finally, we reported on a few other well-known and old drugs that could be repurposed as their indications, doses and side effects are known and thus can be easily managed.

Fluvastatin, rosuvastatin, atorvastatin and simvastatin are statins used to lower the synthesis of cholesterol by inhibiting HMG-CoA. Statins may be an important adjuvant for the treatment of fungal infections because their efficiency has been reported against fungi, even against azole-resistant yeasts [65,92]. Indeed, Macreadie et al. demonstrated a strong inhibition of the growth of *Candida* spp. (with the exception of *C. krusei* on YEPD media containing 100 µM of statins) and *A. fumigatus* [93]. On the fungal cell, statins might work on the pathway of mevalonate synthesis causing a decrease in ergosterol quantity of the cell membrane [93]. Statins showed in vitro synergy with various azoles and may display beneficial outcomes on candidiasis according to one cohort study in 2013 [65,81,94].

Disulfiram is an alcohol antagonist drug that has been used in clinical practice for many years. Khan et al. reported its antifungal potential in 2007 and provided an MIC range from 1 to 16 μg/mL for fluconazole-sensitive and resistant yeast strains, with an inhibiting effect on biofilm formation [95,96]. It also had a fungicidal activity on *Aspergillus* spp. [95]. Earlier, Shukla et al. demonstrated that disulfiram could reverse Cdr1p-mediated drug resistance so it could be used in combination to sensitize resistant strains [63,64]. Broadly, side effects of disulfiram are uncommon, contraindications include pregnancy and unstable cardiovascular disease and close hepatic monitoring is required [97]. Given all this information, in vitro, in vivo and clinical trials should be pursued to fully justify the repositioning of this molecule.

## 4. Some Emerging Multidrug-Resistant Fungi and Their Compounds with Repurpose Potential Identified through Phenotypic Screening

**Figure 2:**
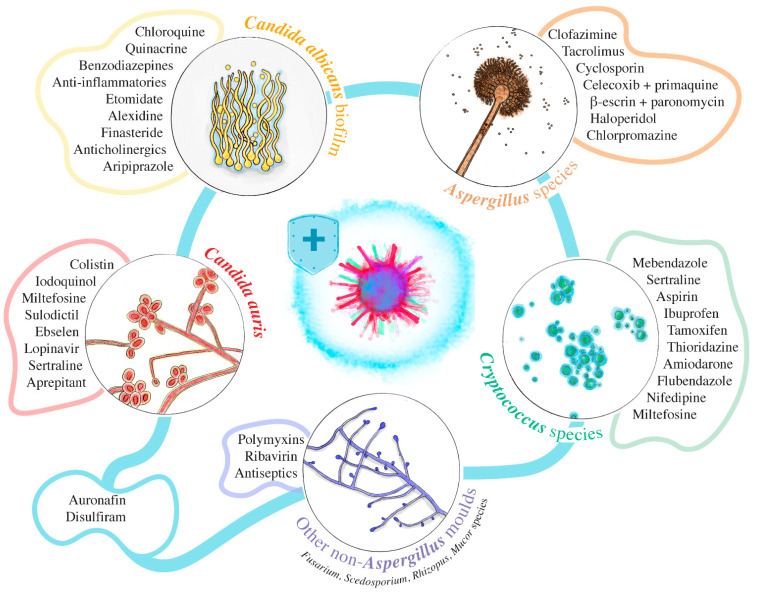
Examples of approved and candidate drugs with activity against emerging MDR fungi.

Resistance to at least one class of antifungal agents is a concern of most existing fungal species. However, some major pathogens have a relatively high resistance rate and constitute a serious public health burden, especially *C. albicans*, *Cryptococcus* spp. and *Aspergillus* spp. In addition, other emerging and potentially life-threatening pathogens are increasingly being reported [98]. Some are still not well characterized and are opportunistic and MDR, such as *C. auris* [99], *Scedosporium* spp. and *Fusarium* spp. [100]. We present a non-exhaustive list of compounds below that could be effective as an alternative therapeutic strategy, depending on these MDR species (Figure 2).

### 4.1. C. albicans Biofilms

The pathogenicity of the *Candida* species resides in their ability to form biofilms, thereby protecting them from external elements such as antifungal agents or the host’s immune system. The common implantation of a *Candida* biofilm on a medical device such as a catheter can lead to candidemia and severe systemic infections. In the absence or failure of treatment, the health and economic consequences are significant [101]. Some drugs alone or in combination with a currently used antifungal are effective in vitro to reduce or inhibit the formation of a biofilm [102,103].

As reported above, chloroquine reverts the azole resistance in biofilms [46], and aspirin [104], disulfiram [96] or auranofin [78] decrease in vitro the biofilm formation of many *Candida* species. Quinacrine is an antimalarial, which was used during WWII but remains available to treat giardiasis or cutaneous leishmaniasis. It has been effective at preventing and treating *C. albicans* biofilms with MIC ranges of 64–256 µg/mL [93]. In addition, both amphotericin B and caspofungin have synergy with quinacrine with an FICI equal to 0.37 and 0.31, respectively [105]. Benzodiazepines such as midazolam or diazepam have also been proposed to be repurposed against the biofilm formation of yeast, due to interaction with its virulence factors [106,107]. Anti-inflammatory compounds appear to be very efficient, such as celecoxib, etodolac, meloxicam, etc. [81]. In addition, flufenamic acid (a nonsteroidal anti-inflammatory drug, NSAID) showed an excellent action in the prevention and treatment of biofilms from echinocandin-resistant strains [108]. The hypnotic agent, etomidate, works against the biofilm of fluconazole-resistant *Candida* spp. strains [109]. The antiseptic alexidine [103] and the finasteride used to treat the prostatic hyperplasia [110] were highly active in vitro at preventing *C. albicans* biofilms. Nile et al. highlighted the cholinergic receptor, which slowed down the biofilm-mediated virulence of *C. albicans* while having a boosting effect on the host immune response using pilocarpine [111] or tropicamide in a study by Machado et al. [112]. The antipsychotic drug aripiprazole was as effective as azoles by acting on the early formation of the pseudo hyphal [113]. As mentioned, many drugs from different therapeutic classes can be effective as an antibiofilm agent. In vivo studies first, followed by clinical trials remain important to confirm those in vitro options.

### 4.2. C. auris

*C. auris* is considered to be a serious global health threat due to its association with nosocomial invasive infections, its high mortality rate and its multidrug resistant profile [114]. High rates of antifungal resistance have been reported for fluconazole and amphotericin B, which is considered to be an intrinsic resistance, but some acquired resistances to echinocandins have been described in some countries [115,116]. The need for effective agents must be a priority in order to address future outbreaks, which, given the current situation, are highly likely [117]. To quickly identify candidate drugs, screening molecule libraries can offer various solutions [49,66,118]. Cheng et al. found six novel anti-*C. auris* compounds among more than 4300 approved drugs with 13 possible different drug associations [118]. The amebicide iodoquinol and leishmanicide miltefosine were reported in those screenings as being potential repositionable compounds due to their inhibition of *C. auris* growth [49]. Sulodictil [66], ebselen [66], antiemetic aprepitant [119] and lopinavir [120] were active against *C. auris*, either alone or in association with currently used antifungals. Finally, most of the drugs described above as being effective on yeast are also effective on *C. auris*, such as sertraline [121], oxyclonazide [44], colistin [112], etc. Indeed, these molecules are generally active via novel targets, which are still naïve in antifungal treatments and therefore in yeast adaptation.

### 4.3. Aspergillus Species

Invasive aspergillosis threatens the lives of millions of immunocompromised patients every year, with a mortality rate of 50/60% [98]. These species come from the environment and some patients are infected via contaminated foods [122,123]. Used for agricultural production, certain fungicides display a similar mechanism as azoles used in clinical routine. The evolution of resistance by selective pressure is attributed to their widespread use and there is an urgent need to find alternative strategies to current antifungals [124,125,126]. As a result, different drug screenings have been performed and have found that clozafimine, tacrolimus, cyclosporin [127], haloperidol [79], disulfiram [95], chlorpromazine [58] and auranofin [122] as reported above, could be repurposed as an anti-*Aspergillus* spp. treatment. Interesting synergies were also obtained between celecoxib and primaquine and paromomycin and β-escin with a FICI of <0.27 or <0.38, respectively, against *A. fumigatus* growth [128].

### 4.4. Cryptococcus Species

Occurring predominantly during the course of an immunocompromised patient’s disease, cryptococcosis causes pneumonia or meningoencephalitis, due to an inhalation of *Cryptococcus* cells from the environment [129]. In addition to their worldwide spread, treatment of these infections remains challenging for clinicians who deal with only three classes of antifungal agents, as echinocandins are not effective against them [89]. Several molecules were found to be active against *Cryptococcus* cell growth, as mentioned above, such as auranofin, aspirin, ibuprofen [122], tamoxifen [129], mebendazole [48], sertraline [86,121] and disulfiram [95], and thioridazine, amiodarone [122], miltefosine [130] and calcium channel blockers such as nifedipine [131] have also been described. The antiparasitic drug, flubendazole, was very active, with MICs ranging from 0.039 to 0.156 μg/mL, even against fluconazole-resistant strains [131]. Despite this, the perfect anticryptococcal treatment would exhibit a low toxicity for polymedicated, immunosuppressed patients would be well-distributed around the body to eradicate all cryptococcal niches including the cerebrospinal fluid [129].

### 4.5. Other Non-Aspergillus Molds

More rarely, other MDR fungal pathogens can cause aggressive and disseminated infections associated with poor prognosis such as Hyalohyphomycetes, including the genera *Fusarium*, *Scedosporium*, etc., or the Mucormycetes with *Mucor* and *Rhizomucor* group. Early and effective treatment is required to prevent the progression of the infection and to limit outbreaks [132,133]. However, this infection remains difficult to treat because the aforementioned fungi are resistant to most current antifungal drugs (Table 1) [132,134]. We previously screened a library of 1280 drugs against six of these filamentous fungi including *Fusarium, Scedosporium, Rhizopus* and *Lichtheimia* species [135]. The main hits found were antifungals, antiseptics and some antineoplastics against a few strains, and polymyxins [41], disulfiram [95], auranofin [122] and ribavirin [68], as mentioned above. These fungi have fewer hits and further investigations are warranted in order not to reach fatal therapeutic impasses. Some combinations of antibiotics and antifungals, however, displayed strong synergies [65].

## 5. Further Assessment and Prioritization of Repurpose Potential

It is important to highlight in this effort that preliminary in vitro studies, however, encouraging, do not automatically imply repurpose potential. In addition, not all compounds identified above possess equal repurpose potential while others may be altogether inappropriate for the clinical use in question. Given that safety data for marketed drugs and advanced clinical candidates are available, further assessment should be undertaken to evaluate and prioritize each molecule with respect to its repurpose potential. For example, effective auranofin concentrations that inhibited yeasts and molds’ growth, ranged from 0.25 to 16 µg/mL [50], but human pharmacokinetics displayed a *Cmax* of 0.025 μg/mL after 6 mg per day [136]. Aspirin on the other hand was effective against yeasts [44,69] in accordance with human doses and used for a long time now. For analgesic dosages, the *Cmax* was reported on average at 50 µM (9 mg/L) and were higher for anti-inflammatory use [137]. Another example, quinacrine alone, was effective against *C. albicans* biofilms with MICs between 64 and 256 µg/mL [63]. However, quinacrine pharmacokinetics from an intrapleural dose displayed a *Cmax* below 1 µg/mL for a 600 mg dose [138] and the therapeutic dose is initially 100 mg, which makes the use of this molecule in monotherapy inappropriate. In spite of that, its use in synergic combinations led to a reduce in the initial dose, from 64 µg/mL alone to 4 µg/mL with caspofungine [105] and approaching acceptable human concentrations. At the current stage, the use of quinacrine as a monotherapy does not reach the reported human concentrations, but the study of its combinations with other molecules is still interesting in view of the reported synergisms. Plasma concentrations of synergistic combinations of azoles or amphotericin B and colistin can be above some previously reported MICs, where human doses of colistin have resulted in serum concentrations up to 32 µg/mL [41]. In such cases, the renal condition of the patient must be considered, although nebulized doses of colistin could avoid this toxicity [139]. Therefore, an analysis process must be applied to all molecules before their repurposing after in vitro, in vivo and other assays. In summary, strict and careful analysis is essential before administration, concerning the consistency between the effective dose and the serum concentration/toxicity dose, but also covering all other parameters related or not to the molecule, such as potential drug interactions, bioavailability of the molecule, strain susceptibility, patient condition and consent, approval of the patient’s care panel, etc.

## 6. Conclusions

The emergence of new mycotic agents and the increase in antifungal resistance has led to the need to find new and/or alternative drugs. We are currently seeing that non-traditional antimicrobial agents, previously prescribed to treat non-infectious conditions, may display antimicrobial properties, and it would be a worthwhile investment to further explore these compounds before being repurposed. Otherwise, these reported drugs could serve as a starting point for the reinnovation of a new molecule. We noted compounds ranging from anti-inflammatory to antipsychotic drugs, which have been documented to control fungal growth and to be repositionable. However, their clinical application may be limited to treat life-threatening fungi, due to drug toxicity, especially with anticancer drugs that generally target eukaryotic organisms or with drugs inducing an immunosuppressive state. Susceptibility testing on each fungus, careful analysis of pharmacokinetic/pharmacodynamic (PK/PD) data and human achievable and tolerable concentrations must be confirmed before administration to assess the benefit/risk. All effective treatments should be taken into consideration as a last-line therapy, even if they have side effects, if they could save the patient [140]. Fortunately, in in vitro assays some of these compounds act synergistically with currently used antifungal agents. Thus, combinations enable the use of low concentrations with the advantage of minimizing any possible undesired physiological effects. This also enhances the efficiency of traditional antimicrobial drugs, which are fungistatic when used alone under normal conditions.

Assuming that obstacles may be overcome, drug repurposing is a promising alternative strategy into which further research and clinical trials are essential to combat the increase in invasive fungal infections.

## Figures and Tables

**Table 1:** Current main antifungal agents: mechanisms of action, clinical indications, side effects and mechanisms of antifungal resistance. FCZ: fluconazole, ITZ: itraconazole, VRZ: voriconazole.

Antifungal Classes	Mechanisms of Action	Clinical Indications	Side Effects	Mechanisms of Resistance	Common Resistant Species
**Polyenes** *Amphotericin B* *Nystatin*	Ergosterol binding (membrane) permeabilization by ion channel formation Cell content leakage	Invasive fungal infection Topical *Candida* infections	Renal toxicity Hypokalemia Phlebitis Immunoallergic reaction	Deficiencies in *ERG2* and *ERG3* genes Ergosterol synthesis alteration Modifications in membrane sterols Changes of enzymatic activity or signaling pathways	*Scedosporium* spp., *Candida lusitaniae*, *Aspergillus terreus*
**Azoles** *Fluconazole* *Itraconazole* *Voriconazole* *Posaconazole* *Efinaconazole* *Isavuconazole*	Inhibition of lanosterol Ergosterol synthesis inhibition Alteration of fungal membrane fluidity and agility	All invasive candidiasis Cryptococcal meningitis *Aspergillus* spp. infections	Digestive disturbancesCephalgias Hepatotoxicity Drug interactions (CYPP450)	Over expression of efflux pump’s function *ERG11* gene mutations inducing blockage in azoles binding Up-regulation of enzyme target Bypass pathway development by *ERG3* gene mutation	FCZ: *Candida krusei*, *Aspergillus* spp., *Scedosporium* spp.*, Fusarium* spp., Mucorales ITZ: *Fusarium* spp. VRZ: Mucorales
**Echinocandins** *Micafungin* *Caspofungin* *Anidulafungin*	Inhibition of β-1,3-glucan synthase (β-GS) Formation of a defective cell wall	Invasive candidiasis Invasive aspergillosis (2nd intention)	Good overall tolerance	Mutations on *FKS1* gene (encoding for a subunit of β-GS) Decrease of affinity between drug and target	*Cryptococcus* spp., *Fusarium* spp., *Scedosporium* spp., Mucorales
*5-fluorocytosine*	Nucleoside analogue Disruption of protein synthesis Inhibition of DNA synthesis	Cryptococcosis Invasive candidiasis if treatment failure Always in association	Gastrointestinal troubles Hepatotoxicity Hematotoxicity	Mutations on *FUR1* gene (encoding uracil phosphoribosyl transferase) Mutations on *FCY1* gene (encoding cytosine deaminase enzyme)	Ineffective against many filamentous fungi

## Data Availability

No new data were created or analyzed in this study. Data sharing is not applicable to this article.
